# Prediction of Metabolic Gene Biomarkers for Neurodegenerative Disease by an Integrated Network-Based Approach

**DOI:** 10.1155/2015/432012

**Published:** 2015-05-03

**Authors:** Qi Ni, Xianming Su, Jingqi Chen, Weidong Tian

**Affiliations:** ^1^State Key Laboratory of Genetic Engineering, Department of Biostatistics and Computational Biology, School of Life Sciences, Fudan University, Shanghai 200438, China; ^2^Department of Geriatric Cardiology, The First Affiliated Hospital of Xi'an Jiao Tong University, Xi'an, Shaanxi 710000, China

## Abstract

Neurodegenerative diseases (NDs), such as Parkinson's disease (PD) and Huntington's disease (HD), have become more and more common among aged people worldwide. One hallmark of NDs is the presence of intracellular accumulation of specific pathogenic proteins that may result from abnormal function of metabolic processes. Previously, we have developed a computational method named Met-express that predicted key enzyme-coding genes in cancer development by integrating cancer gene coexpression network with the metabolic network. Here, we applied Met-express to predict key enzyme-coding genes in both PD and HD. Functional enrichment analysis and literature review of predicted genes suggested that there might be some common pathogenic metabolic pathways for PD and HD. We further found that the predicted genes had significant functional association with known disease genes, with some of them already documented as biomarkers or therapeutic targets for NDs. As such, the predicted metabolic genes may be of use as novel biomarkers not only for ND diagnosis but also for potential therapeutic treatments.

## 1. Introduction

Neurodegenerative diseases (NDs), such as Parkinson's disease (PD) and Huntington's disease (HD), occur as a result of progressive loss of structure or function of neurons [1]. Onset of NDs is generally for people at old age, but symptoms can appear at any age. ND patients often have difficulties with motor or cognition. Currently, PD affects about 0.16% of the general population and up to 2% among people above 80 years old. Symptoms of PD conventionally include slowness of movement, unrest shaking, rigidity, and postural instability [2]. PD can also cause ruddy and oily skin, depression mood, and dysautonomia problems [3]. The prevalence of HD is about 5 affected individuals per 100 000 population, with a higher incidence rate in white population compared with Asia or African people [4]. Individuals with HD usually show distinct chorea and motor impersistence and may have psychiatric disturbances [4, 5]. Most patients start presenting cognitive decline at early stage resulting in dementia in late stage [5]. Both of these disorders bring serious disability to the patients and cause significant burden to their families. Many researchers have worked on therapies for NDs for a long time. However, the precise pathologies of NDs are still poorly understood and effective therapies are still needed to be explored.

The pathogenesis of NDs is usually associated with several interacting pathways, which finally coincide into a common pathway of apoptosis [6]. Changes in membrane phospholipid molecules were implicated in cell death [7]. Mitochondrial dysfunction, one of the most studied pathways, can result in oxidative stress and energy supply abnormality for neuronal cells [8]. Immune pathways, such as inflammation progress, lead to oxidative stress and cause apoptosis through more direct mechanisms [9]. In addition, accumulation of abnormal proteins is a typical symptom of many NDs and has been thought to be a key factor in the pathogenesis for a long time. Each disorder has specific protein aggregates as its hallmark: *α*-synuclein in the substantia nigra of PD and huntingtin in the striatum of HD [1]. Abnormal protein accumulation can result from misfolded proteins of the synthesis pathways or toxic metabolites associating with the protein synthesis pathways. Genetic mutations explained some familial form cases, but many sporadic cases were caused by unclear reasons [10]. Mutations of protein coding genes* PARK2* and* HTT* are culprits of PD and HD [1], respectively. The expansion of trinucleotide CAG repeats coding for polyglutamine in the N terminus of the huntingtin protein can cause HD. When the CAG repeats reach 36, the disease is penetrant [4]. Oxidation products of proteins, such as advanced glycation end-products (AGEs) and the deposition of AGEs-cross-linked proteins, exist in many NDs [11]. Oxidative modifications, nitration, and phosphorylation of proteins were also suggested to be involved in the aggregation of pathogenic proteins [1]. These discoveries lead to the common hypothesis that the misfolding and accumulation of specific proteins are pivotal to ND pathology.

The accumulation of misfolded proteins in ND patients is associated with abnormal metabolic conditions [11–13]. Detection of enzyme-coding genes that are significantly differentially expressed and that may drive the metabolic changes in ND patients may therefore help understand the mechanisms of ND development. In a previous study, we have developed a computational method named Met-express that integrated cancer gene coexpression network and metabolic network to predict key enzyme-coding genes in cancer development [14]. In Met-express, a coexpression network of cancer gene was first constructed, from which cancer-specific gene coexpression modules were determined. Then, within a cancer-specific gene coexpression module, Met-express was aimed at identifying the enzyme-coding genes that were coexpressed with significantly more metabolite-sharing enzyme-coding genes. The hypothesis was that these genes likely bear a higher potential to alter the metabolism of cancer cells and therefore might serve as anticancer drug targets. We demonstrated with both literature search and real experiments to show that the predicted enzyme-coding genes by Met-express in cancer were critical for cancer development. As a general method, Met-express can be extended for predicting key enzyme-coding genes in other diseases, such as NDs. Thus, we applied Met-express to both PD and HD and have predicted a number of key enzyme-coding genes for the diseases. Functional enrichment analysis and literature validation of predicted genes suggested that there might be some common pathogenic metabolic pathways for PD and HD. In addition, predicted genes were found to have significant functional associations with known ND genes. As such, these genes may not only help to understand the mechanisms of ND pathogenesis but also be used as biomarkers for ND diagnosis and for developing novel therapeutic strategies.

## 2. Materials and Methods

### 2.1. Microarray Data Preparation

All microarray data were downloaded from GEO database (http://www.ncbi.nlm.nih.gov/geo/). For PD, GDS files were downloaded only if the dataset (i) contained samples from brain tissues of both normal people and patients, (ii) had more than 10 samples, and (iii) had not been treated with any stimulus. As for HD, GDS files were also following the three standards except that they were from blood tissues. These resulted in four GDS files of PD and three of HD. As two files of PD were from the same samples and tested on similar microarrays, we combined the two files to one file to extend the data size (Supplementary Table 1in Supplementary Material available online at http://dx.doi.org/10.1155/2015/432012). For each GDS file, we selected only genes with a median ratio of null values less than 80% and used R (http://www.r-project.org/) to normalize gene expression values as previously described [14].

### 2.2. Met-Express Procedure

We followed the procedures as Chen et al. described [14] to detect the key enzyme-coding genes of PD and HD. Here, we briefly describe the workflow of Met-express. (1) We calculated Pearson correlation coefficients (PCC) based on expression levels for all pairs of genes and used a network partition algorithm named Qcut to divide gene coexpression network into gene coexpression modules, retaining modules containing more than 10 genes. Then, we evaluated the specificity of each coexpression module to the disease conditions by computing the AUC of a ROC curve plotted using the median expression value of the genes inside the module. (2) We extracted the information of reactions, corresponding enzyme-coding genes, and associated metabolites from the KEGG Markup Language files (http://www.genome.jp/kegg/). Two enzymes were linked if the substrate metabolite of one enzyme was the product metabolite of the other. The direction was defined as starting from the enzyme generating the metabolite to the enzyme consuming it. Following this rule, we constructed the metabolic network by linking enzymes, when the corresponding metabolite participated in fewer than 18 reactions and not in “Xenobiotics Biodegradation and Metabolism.” In the construction process, a reversible reaction would be split into two reactions. The above procedures resulted in 860 enzyme-coding genes with outward edges, which were considered to have large impacts on the metabolic network. (3) Finally, we computed the importance score of each gene by considering both specificity of the gene coexpression module to the disease and enrichment of the degree of metabolic links of the gene inside the module. For each dataset, we used the median importance score as the cutoff to select the candidate genes. Then, those genes predicted in at least 2 datasets were taken as the key enzyme-coding genes for the disease.

### 2.3. Functional Enrichment Analysis

Gene ontology (GO) enrichment analysis and KEGG pathway enrichment analysis were both conducted by Fisher test using fisher.test in R. *P* value was adjusted by false discovery rate. All the enzyme-coding genes in the constructed metabolic network were used as the background.

### 2.4. Functional Association Test

To test the connection between our predicted key enzyme-coding genes and known disease genes in database, we calculated the shortest path lengths between each pair of genes in a functional association network, specifically the FunCoup [15] network. Here, the shortest paths were calculated by Dijkstra's algorithm [16]. For each identified enzyme-coding gene of each disease, we calculated the mean shortest path between the key enzyme-coding gene and all the known disease genes in DrugBank or HGMD. The mean distance for all key enzyme-coding genes was adopted as the distance between our identified genes and known disease genes. As a comparison, we randomly selected the same number of genes as that of the predicted enzyme-coding genes and repeated the above procedures 1000 times. The probability of the case that the mean shortest path length for random selected gene was less than or equal to that of our identified genes was used as the *P* value.

## 3. Results and Discussion

### 3.1. A Brief Introduction of Met-Express

**Figure 1:**
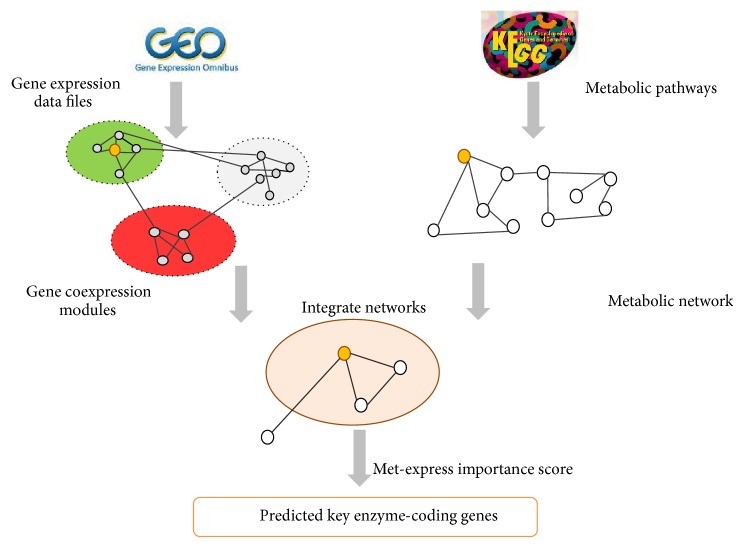
The procedure of Met-express. In the gene coexpression modules part, red represents upregulated modules, green represents downregulated modules, and grey means that genes in the module do not change much in expression. For detailed procedures please refer to Section 2, and the methods in Chen et al. [14].

Met-express is a published method originally applied to cancer expression data. It has been successfully used to predict key enzyme-coding genes as potential candidates for therapeutic uses [14]. As in Figure 1, it integrated disease specific gene coexpression network and the metabolic network, to identify those enzyme-coding genes that have a potential to influence downstream genes and thus may play an important role in the disease-related pathways.

### 3.2. Application of Met-Express to Predict Key Enzyme-Coding Genes in Neurodegenerative Diseases

We constructed a metabolic network with 860 enzyme-coding genes and then applied Met-express to predict key enzyme-coding genes in each of the selected ND datasets. For both PD and HD diseases we combined those genes predicted in at least two of the disease-related datasets and considered them candidate key enzyme-coding genes to the respective diseases.

**Figure 2:**
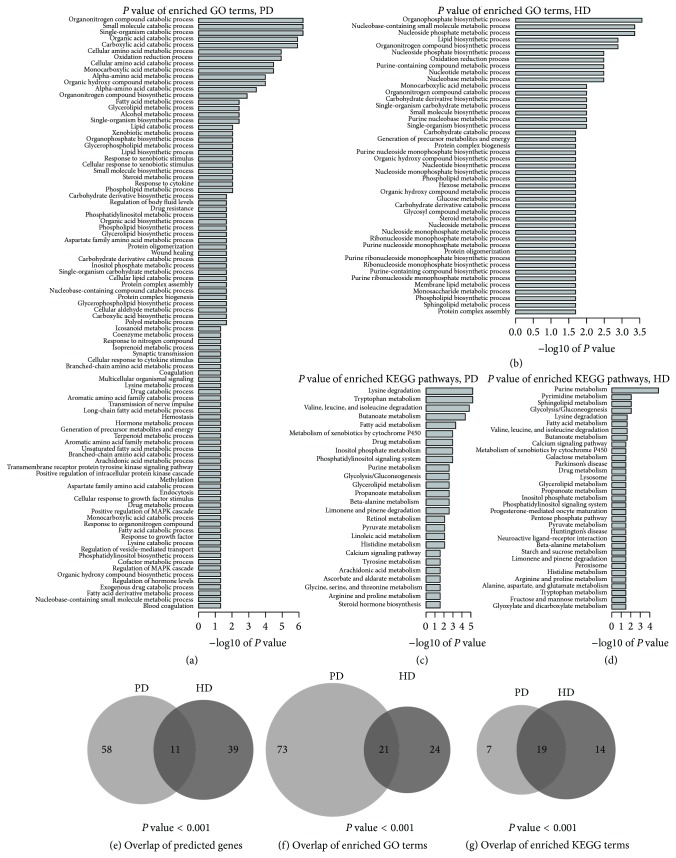
(a) and (b) The −log 10 based adjusted *P* value of significantly enriched GO terms (FDR adjusted *P* values ≤ 0.05) for predicted genes in PD (a) and HD (b). (c) and (d) The −log 10 based adjusted *P* value of significantly enriched KEGG terms (FDR adjusted *P* values ≤ 0.05) for predicted genes in PD (c) and HD (d). (e) The number of overlapped genes between the predicted key enzyme-coding genes of PD and HD. (f) and (g) The number of overlapped significantly enriched GO terms (f) and KEGG terms (g) for predicted key enzyme-coding genes of PD and HD.

We applied Met-express to three datasets (GDS2821, GDS3128_GDS3129, and GDS4154) related to PD and predicted 118, 164, and 114 key enzyme-coding genes, respectively. The predicted genes from GDS2821 and GDS3128_GDS3129 shared 35 genes (*P* value = 0.007), while the number of shared genes for GDS2821 and GDS4154 and GDS3128_GDS3129 and GDS4154 is 15 and 33 (*P* value = 0.012), respectively. In total, the number of genes predicted in at least two datasets was 69, and we considered them key enzyme-coding genes for PD (*P* value = 0.013, Supplementary Table 2). Seven out of these 69 genes were predicted in all the three datasets (*P* value = 0.040). Three microarray data of HD, GDS1331, GDS1332, and GDS4541, were analyzed and resulted in 114, 155, and 79 predicted genes, respectively. There were 50 genes predicted in at least two datasets, and they were considered key enzyme-coding genes for HD (Supplementary Table 2). Four of the 50 genes were predicted in all the three datasets. Among the predicted key enzyme-coding genes in PD and HD, 11 genes were detected in both diseases (Figure 2(e)). However, the 11 genes showed different expression patterns. Among them, only ALDOA and PFKM had consistent expression pattern and were downregulated in both diseases. CYP2B6 and GALC were upregulated in PD and had different expression pattern in different HD datasets. The remaining 7 genes were expressed differently in different datasets. These showed the complex mechanism involved in the pathogenesis of NDs.

Functional enrichment analysis of predicted genes resulted in 94 GO terms and 26 KEGG pathways enriched for PD genes and 45 GO terms and 33 KEGG pathways enriched for HD genes (Figures 2(a), 2(b), 2(c), and 2(d)). The enriched GO terms and KEGG pathways covered various biological processes including “oxidation reduction process,” “lipid biosynthetic process,” “purine metabolism,” and “tryptophan metabolism.” Among the enriched terms of these two diseases, 21 GO (*P* value < 0.001) terms and 19 (*P* value < 0.001) KEGG pathways were detected in both diseases (Figures 2(f) and 2(g)). The significant functional overlaps between PD and HD genes indicated that there might be common mechanisms in the pathogenesis of NDs. The common pathways included “protein oligomerization,” “lipid biosynthetic process,” and “phosphatidylinositol signaling system,” which have already been suggested to play critical roles in the development of NDs. For example, the accumulation of *α*-synuclein oligomers was found in PD patients [17], while, in cell line and mouse HD models, the accumulation of intracellular polyglutamine oligomers was suggested to be directly related to HD phenotypes [18]. Cholesterol biosynthesis, one of the most important lipid biosynthetic processes in brain, has been found to be significantly altered with decreased activity in both Parkinson cell lines [19] and HD patients' blood [20]. Alterations in the phosphatidylinositol signaling pathway have also been observed in both cell line models and ND patients and have been linked to autophagy disruption in the pathology of PD and HD [21–23]. The above evidence supported our predictions that alteration and disruption of these pathways may be common features of NDs. Hence, enzymes and metabolites predicted in these pathways may serve as biomarkers for NDs, and targeting on the common metabolic pathways may effectively help with treatment of these diseases.

### 3.3. Predicted Genes Were Significantly Associated with Known Disease Genes from DrugBank and HGMD

**Figure 3:**
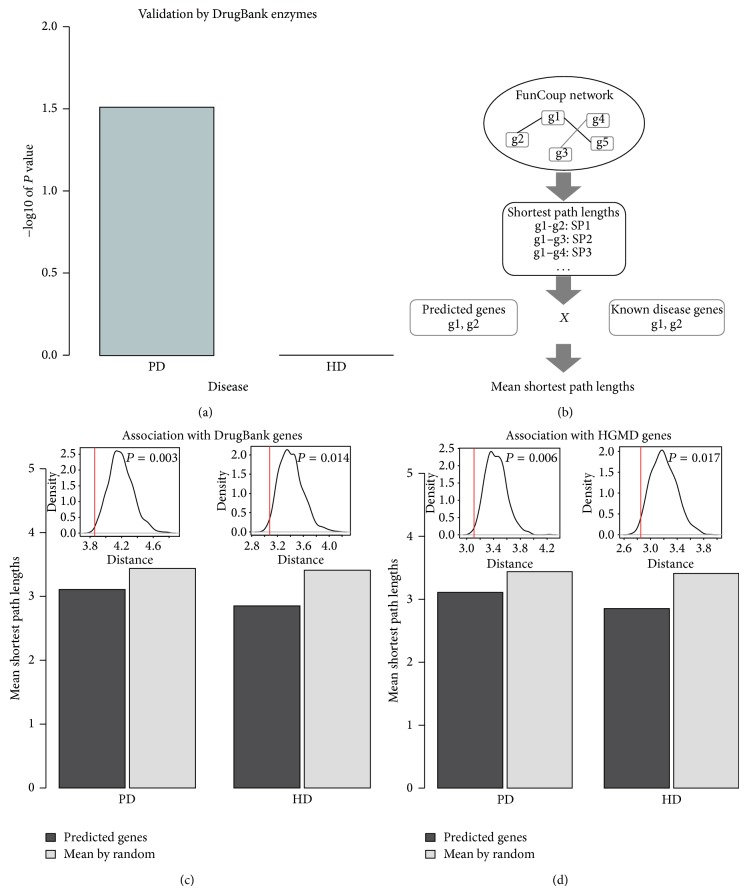
(a) The −log 10 based *P* value of enrichment of predicted key enzyme-coding genes of PD and HD in DrugBank enzymes for the two diseases, respectively. (b) The flowchart of how to calculate the mean shortest path lengths between predicted enzyme-coding genes and known disease genes from public databases. (c) and (d) The mean shortest path lengths of predicted enzyme-coding genes for PD and HD with disease genes from DrugBank (c) and HGMD (d) (the bars, compared with random), and the corresponding distributions of the mean shortest path lengths of random selected genes (the line plots above the bars). The red lines marked the actual mean shortest path lengths for predicted enzyme-coding genes with known disease genes. The black lines represented the distributions of the mean shortest path lengths of random selected genes with known disease genes.

A few of our predicted enzyme-coding genes have already been recorded as biomarkers or drug targets in current known databases: four of the key enzyme-coding genes of PD have been recorded in DrugBank [24] database (http://www.drugbank.ca/), which function in catalyzing PD drug-related reactions (*P* value = 0.0308), while no key enzyme-coding genes of HD have been recorded in this database (Figure 3(a)). The low overlap between our predicted genes and DrugBank database may be due to the relatively small number of known disease-related enzyme genes. To further investigate the functional association of the predicted genes with known disease genes, we calculated the gene-gene shortest path using the FunCoup [15] network.

The idea is that if a predicted gene is strongly functionally associated with a known disease gene, we would expect to see that they are close to each other in the network; that is, they have significant shorter shortest-path length in the network than random genes. Here, for each disease, the mean shortest path lengths between our predicted genes and the known disease genes in DrugBank or HGMD [25] databases were computed as an evaluation of functional association (Figure 3(b)) and compared to the mean shortest path lengths between random sets of genes and known disease genes. As illustrated in Figures 3(c) and 3(d), the mean shortest path lengths of PD key enzyme-coding genes with two lists of disease genes were both significantly shorter than random (both *P* value < 0.01). The mean shortest path lengths of HD key enzyme-coding genes with two database disease genes were also both significantly shorter than random (*P* value < 0.05). Besides DrugBank and HGMD, we have compared our predicted genes to OMIM [26] (data not shown because of too few genes documented for HD) and GAD [27] (Supplementary Figure 1) databases with functional association test and validated that our predicted genes were also significantly functionally associated with the known disease genes in these two databases. These results indicated that our identified key enzyme-coding genes were strongly functionally associated with known disease genes and might participate in the related processes of known disease genes.

### 3.4. Some Predicted Enzyme-Coding Genes Have Been Implicated to Function in NDs

Besides validation with known databases, literature data mining was also performed for some of the predicted genes. Here we present several examples.

*ALDH2* is one of the predicted genes for PD. It encodes mitochondrial aldehyde dehydrogenase 2, which participates in “Glycolysis/Gluconeogenesis,” “fatty acid degradation,” and many other amino acid metabolic pathways [28]. The activity of mitochondrial aldehyde dehydrogenase 2 was found significantly to be increased in the putamen of PD patients compared to controls [29], and this gene was recently recommended as a new therapeutic target for PD [30].* MTHFD1*, another predicted key enzyme-coding gene for PD which encodes a protein that possesses three distinct enzymatic activities, has been suggested to be involved in PD mechanisms through homocysteine catabolic process [31].

There are also a number of predicted genes that have been reported in ND-related pathways. For example,* ALDOA* has the most significant association with known disease genes of both PD and HD based on the mean shortest path calculation (Table 1). This gene is downregulated in both PD and HD and was found to be associated with PD-pathogenic proteins *α*-synuclein and DJ1 in mouse cells [32] and significantly downregulated in the mitochondrial fraction of PC12 cells after PD-linked compound dopamine treatments [33].* TDO2*, another predicted gene for PD, encodes a critical enzyme, tryptophan 2,3-dioxygenase, in the kynurenine pathway of tryptophan metabolism [34]. This enzyme initiates the metabolism of tryptophan by catalyzing tryptophan to kynurenine, which then is metabolized in two alternative ways either to kynurenic acid or to 3-hydroxykynurenine, both neurotoxic. In PD patients, kynurenine/tryptophan ratio was reported to be higher than in healthy people [35], and the level of kynurenic acid was lower in postmortem analyses of HD patients' brains than control [36]. These indicated that the kynurenine pathway of tryptophan metabolism may be associated with the pathogeneses of both PD and HD and that* TDO2* can be used as a biomarker or therapeutic target for these two diseases.

We have also predicted some novel key genes for NDs, which have little or no previous related reports. Some of these genes function in important pathways of NDs. For example,* AACS*, one of the 11 predicted genes in both two diseases, encodes an enzyme catalyzing cholesterol synthesis and fatty acids synthesis with high expression in human brain [37]. It is important for neurite outgrowth, and knockdown of* AACS* has been reported to influence the expression of some neural marker, such as synaptopodin and MAP2 [38].* SGMS1* encodes the Golgi isoform of sphingomyelin synthase. Its overexpression was associated with suppression of ceramide production and apoptosis after photodamage [39].* SPTLC1* encodes a subunit of serine palmitoyltransferase, which catalyzes the rate-limited step of sphingolipid synthesis. Mutation in* SPTLC1* would cause neuronal dysfunction [40]. These results implied that* AACS*,* SGMS1*, and* SPTLC1* might contribute to the developments of NDs via lipid metabolic process and oxidation reduction process. Therefore, our predicted key enzyme-coding genes may serve as possible new candidates for further researches of ND mechanisms and therapies.

### 3.5. Discussion

Met-express integrates gene coexpression network with metabolic network to predict the primary enzyme-coding genes that may be critical for the development of NDs. The predicted genes and enzymes may influence multiple pathways and can bring important influences for ND-related mechanisms, some of which may help explore the common mechanisms of NDs. For example, as previously discussed,* ALDH9A1* was predicted as key gene for both PD and HD by Met-express. It functions in many important pathways, including Glycolysis/Gluconeogenesis, pyruvate metabolism, arginine and proline metabolism, histidine metabolism, tryptophan metabolism, beta-alanine metabolism, and glycerolipid metabolism [28], all of which were enriched in both PD and HD in our analysis. Another example is* EHHADH*, which was also predicted for both diseases. It functions in pathways such as tryptophan metabolism, beta-alanine metabolism, butanoate metabolism, and fatty acid metabolism, which were enriched by both PD key enzyme-coding genes and HD enzyme-coding genes. Although there have been few published evidences relating* ALDH9A1* and* EHHADH* to PD or HD presently, their roles in the many common pathways between PD and HD make them possible targets for further mechanistic investigations.

Many of the enzyme-coding genes predicted by Met-express encode upstream enzymes in their respective pathways. The abnormal expression of these genes in ND patients may have profound impacts on downstream genes and compounds. As a result, altering the abnormal expression of these genes in ND patients bares the potential to help reverse diseases. For example, as previously analyzed,* ALDH2*, a gene predicted for PD, has been raised by others as a potential therapeutic target for PD [30]. Here, Met-express has provided a pool of candidate genes that are of potential value for further therapeutic investigations.

## 4. Conclusions

Using Met-express, which integrates gene coexpression network and the enzyme network to find key enzyme-coding genes for diseases, we identified 69 and 50 key enzyme-coding genes for Parkinson's disease and Huntington's disease, respectively. Comparison between the functional analyses of the predicted genes indicated that there might be some common pathogenic metabolic pathways for NDs. The predicted genes for PD had a significant overlap with the annotated enzymes in DrugBank. Moreover, predicted genes for PD and HD both showed significantly closer association with known disease genes from DrugBank, HGMD, and other databases than random as evaluated by the mean shortest path lengths. Some of the identified key genes have been reported to be important in the disease-related processes by previous publications. Some novel findings showed potential in influencing pathways that are important in NDs. Thus, application of Met-express to NDs can provide candidates for disease biomarkers and may help with the further research of the etiology, pathology, and therapy of these diseases.

## Supplementary Material

Supplementary Table 1: Selected GEO files for each disease.
Supplementary Table 2: The predicted key enzyme-coding genes of each disease.
Supplementary Table 3: Target genes and associated enzyme-coding genes for PD and HD in DrugBank (sheet "DrugBank") and disease genes for PD and HD in HGMD (sheet "HGMD").
Supplementary Figure 1: The mean shortest path lengths of predicted enzyme-coding genes for PD and HD with disease genes from GAD, and the corresponding distributions of the mean shortest path lengths of random selected genes.

## Figures and Tables

**Table 1:** Predicted enzyme-coding genes with top 10 closest mean shortest paths to known disease genes.

Disease	Gene symbol	Protein annotation
	ALDOA	Fructose-bisphosphate aldolase A
	PFKM	ATP-dependent 6-phosphofructokinase, muscle type
	GOT2	Aspartate aminotransferase, mitochondrial
	ALDH2	Aldehyde dehydrogenase, mitochondrial
PD	HADHB	Trifunctional enzyme subunit beta, mitochondrial
	MTHFD1	C-1-Tetrahydrofolate synthase, cytoplasmic
	ALDH9A1	4-Trimethylaminobutyraldehyde dehydrogenase
	INPPL1	Phosphatidylinositol 3,4,5-trisphosphate 5-phosphatase 2
	ECHS1	Enoyl-CoA hydratase, mitochondrial
	OGDH	2-Oxoglutarate dehydrogenase, mitochondrial

	ALDOA	Fructose-bisphosphate aldolase A
	GPI	Glucose-6-phosphate isomerase
	PFKM	ATP-dependent 6-phosphofructokinase, muscle type
	IMPDH2	IMP (inosine 5′-monophosphate) dehydrogenase 2
HD	GLA	Alpha-galactosidase A
	SPTLC1	Serine palmitoyltransferase 1
	AK1	Adenylate kinase isoenzyme 1
	EHHADH	Peroxisomal bifunctional enzyme
	POLE3	DNA polymerase epsilon subunit 3
	DCK	Deoxycytidine kinase
